# The Gastrointestinal Tract: A Unique Battlefield for Bioengineering Delivery Platforms

**DOI:** 10.3390/bioengineering12121347

**Published:** 2025-12-10

**Authors:** Teng Ma, Siyu Sun

**Affiliations:** Department of Gastroenterology, Endoscopic Center, Engineering Research Center of Ministry of Education for Minimally Invasive Gastrointestinal Endoscopic Techniques, Shengjing Hospital of China Medical University, Shenyang 110004, China; mateng@cmu.edu.cn

**Keywords:** drug delivery systems, digestive endoscopy, bioengineering, gastrointestinal diseases

## Abstract

Traditional drug delivery methods for gastrointestinal diseases, including oral and systemic administration, often suffer from degradation, inadequate mucosal absorption, and off-target toxicity. Consequently, these methods result in low bioavailability and suboptimal therapeutic outcomes for localized conditions such as inflammation and early-stage cancer. This review examines the innovative integration of advanced bioengineering platforms with therapeutic gastrointestinal endoscopy to address these delivery challenges. We concentrate on three principal bioengineered platforms: (1) nanoparticle systems (e.g., lipid, polymeric, and inorganic nanoparticles) designed for localized chemotherapy and theranostics; (2) in situ-forming hydrogels that serve as intelligent wound management materials and sustained drug depots; and (3) drug-eluting and biodegradable stents that convert passive luminal scaffolds into active, long-term drug-releasing devices. An analysis of these platforms demonstrates that their synergy with endoscopy facilitates precise, minimally invasive, and sustained local therapy, potentially transforming the treatment landscape for gastrointestinal diseases such as cancer and inflammatory bowel disease. Additionally, we investigate advanced strategies, including active targeting and stimulus-responsive release mechanisms, to enhance spatial precision. Despite promising preclinical advancements, clinical translation encounters challenges related to long-term biocompatibility, scalable manufacturing, regulatory pathways for drug-device combinations, and cost-effectiveness. Ultimately, the convergence of bioengineering and endoscopy presents significant potential to usher in a new era of precise, localized, and sustained micro-invasive treatments in gastroenterology.

## 1. Introduction

### 1.1. Clinical Challenges of Gastrointestinal Drug Delivery

Drug treatment of gastrointestinal diseases is a fundamental part of modern medicine. However, traditional routes, such as oral and systemic administration, have limitations for treating many localized gastrointestinal conditions [1,2]. The gastrointestinal (GI) tract has an enormous surface area for oral drug delivery, but its intricate structure and dynamic physiological environment pose many challenges for drug delivery, especially for large-molecule drugs. The main anatomical barrier is composed of one layer of epithelium cells, which have tight junctions at their apices, and is the main physical barrier. The physiological environment is strongly regionally heterogeneous: the stomach has acidic pH (pH 1–2) and a lot of digestive enzymes, which can adversely affect acid-labile and protein-based drugs. The pH in the small intestine and colon increases gradually (duodenum pH 6, terminal ileum pH 7.4, rectum pH 6.7), and they contain different hydrolases, forming a complex biochemical barrier [3,4]. Moreover, drug transport times vary across different sections of the digestive tract, and mucus covers the whole lumen, further inhibiting drug–epithelium contact [5]. Oral administration is the most convenient method for drug delivery; however, the oral bioavailability of nucleic acid drugs, such as siRNA and peptide drugs, is typically very low, often below 2% [6,7]. In non-human primates, the relative bioavailability of GalNAc-siRNA ranges from only 1.4% to 2.5% [8]. This low bioavailability is primarily due to enzymatic degradation in the gastrointestinal tract, the mucosal barrier, and the drug’s low permeability [1]. Injecting drugs systemically can bypass the physical and biochemical challenges of the gastrointestinal tract. However, this method results in only a minimal amount of the drug reaching the targeted lesion, with most of it dispersing throughout the body. Consequently, the therapeutic efficacy diminishes, and there is a heightened risk of significant off-target toxicity [9]. For example, immunosuppressants for inflammatory bowel disease may cause bone marrow suppression, and chemotherapy drugs for gastrointestinal tumors may cause toxic side effects, including liver and kidney damage [10,11,12,13]. Thus, precise delivery of therapeutic doses at the right time and location is a key goal in gastroenterology. This requires new methods for local controllable and targeted drug delivery.

### 1.2. Digestive Endoscopy: From Diagnostic Tools to Precision Treatment Platforms

Gastrointestinal endoscopy has evolved from a simple diagnostic tool to a platform for minimally invasive treatments. Pisipati and Pannala also note that therapeutic endoscopy has rapidly become advanced: a series of new technologies, such as third-space endoscopy (e.g., Peroral Endoscopic Myotomy, POEM), Endoscopic Submucosal Dissection (ESD), endoscopic metabolic surgery, and complex interventional procedures guided by Endoscopic Ultrasound (EUS), have proven that endoscopy possesses the extraordinary ability to “reach the lesion directly” [14]. This capability offers a paradigm-shifting opportunity to overcome the aforementioned drug delivery challenges. By serving as a channel for local drug administration, endoscopy can completely bypass the harsh gastrointestinal environment and the first-pass effect, thereby holding the promise of maximizing therapeutic efficacy at the target site while minimizing systemic exposure and toxicity. Its clinical potential is immense: it is conceivable that under the guidance of EUS, high-dose chemotherapeutic drugs could be directly injected into pancreatic tumors, or sustained-release anti-inflammatory drugs could be precisely delivered to the inflamed intestinal segments of patients with Crohn’s disease.

Current endoscopy treatments depend mostly on physical actions like resection, ablation, and dilation [15,16]. These processes are often immediate and disposable, and can lead to poor healing quality, ulcer complications after ESD, local recurrence after tumor ablation, and restenosis after stent placement [17,18,19]. These problems can be resolved by local and continuous drug administration. Therefore, the true next frontier of endoscopy lies in combining its unparalleled spatial positioning capability with advanced bioengineering platforms, enabling it to function as a long-acting and controllable drug reservoir at the disease site.

### 1.3. Objectives and Scope of This Review

This review differentiates itself from prior studies on gastrointestinal (GI) drug delivery and endoscopic techniques by concentrating specifically on the intersection of advanced bioengineering design and clinical endoscopic applications. While the existing literature frequently treats drug delivery systems and endoscopy as distinct domains, our synthesis presents a novel framework that categorizes and critically assesses platforms based on their compatibility and synergy with endoscopic procedures. We extend beyond a general overview of nanomedicine or hydrogels to elucidate how specific design principles—such as injectability, in situ gelling, and stent-based deployment—can be utilized to address clinical challenges encountered during endoscopy, including post-resection ulcer healing, stricture management, and localized tumor therapy (Table 1). By tracing the pathway from platform design to endoscopic implementation and emphasizing the critical translational challenges inherent to this interdisciplinary approach, this review provides a forward-looking perspective aimed at bridging the divide between bioengineering innovation and practical clinical application in gastroenterology.

## 2. Three Major Drug Delivery Systems

With the in-depth advancement of research, a variety of drug delivery platforms have emerged. However, in this article, we only discuss three types of drug delivery platforms, namely nanoparticles, in situ hydrogels, and stents (Table 2).

### 2.1. Nanoparticle Systems: Precise Intervention at the Cellular and Subcellular Levels

Nanotechnology is a novel approach to drug delivery problems in the digestive tract. Nanoparticles (NPs) are designed materials with four properties: surface and material properties, structure, targeting ability, and responsiveness. By arranging and combining these properties, NPs can be adapted into customized nanoplatforms for targets [38]. For example, nanoparticle size can influence transport in the body. By controlling the size of nanoparticles, their behavior can be “programmed” to increase drug delivery to target tissues and control clearance via the kidneys or liver [39]. To prevent drug degradation in the gastrointestinal tract, pH-responsive nanocarriers encapsulate drugs and release them in the intestine’s alkaline environment, thereby avoiding destruction by gastric acid [2,40]. To address the mucus barrier, nanoparticles modified with polyethylene glycol (PEG) or mucin-penetrating peptides reduce mucus retention and enhance permeability [38]. Milk-derived extracellular vesicles function as a bionic nanosystem, characterized by their natural phospholipid bilayer. This structure protects encapsulated drugs from enzymatic hydrolysis and enhances absorption by intestinal epithelial cells through membrane fusion. Consequently, these vesicles significantly improve the delivery of biological macromolecules, such as insulin and nucleic acids [41]. These strategies synergistically overcome multiple physiological barriers in the gastrointestinal tract. With digestive endoscopy, targeted injection and nanoparticle spraying can bypass the complex physiological barriers of oral administration and avoid the toxic side effects of systemic administration. In this section, we present three promising nanoparticle platforms for gastrointestinal disease treatment: lipid nanoparticles, biodegradable polymer nanoparticles, and organic nanoparticles, with their unique advantages integrated with endoscopic platforms.

#### 2.1.1. Lipid Nanoparticles: Ideal Carriers for Nucleic Acid Drugs

Lipid nanoparticles (LNPs) are the most promising non-viral gene delivery platforms, transporting nucleic acid drugs such as mRNA, siRNA, DNA, and gene-editing tools [42]. The safety and effectiveness of LNPs were demonstrated in the COVID-19 vaccine [43,44,45]. The functions of LNPs depend on the precise interaction of four components. Ionizable lipids are the delivery components, enabling nucleic acid binding and endosomal escape by a pH-responsive mechanism. Phospholipids are the structural framework, providing a stable lipid bilayer and regulating membrane fluidity. Cholesterol acts as a membrane stabilizer, promoting stability and rigidity by filling molecular gaps. PEG-modified lipids prevent particle accumulation and extend circulation time by surface modification [46]. Optimizing the ratio of these components is essential for delivery efficiency and achieving specific tissue targeting.

LNPs are promising carriers of nucleic acids for treating gastroenterological diseases. Many studies show that LNPs can be effective in treating gastrointestinal diseases. In NASH-HCC, Wang showed that YTHDF1 recruits myeloid-derived suppressor cells (MDSCs) via the EZH2-IL-6 direction, inhibiting CD8 + T cell function. By encapsulating YTHDF1 siRNA in LNPs targeting the liver, they down-regulated IL-6 secretion and decreased MDSC infiltration, significantly improving the anti-tumor effects of anti-PD-1 treatment [20]. Bai used LNPs to deliver a CRISPR-Cas9 system knocking Rubicon in a NAFLD mouse model, and reduced lipid deposition in the liver and increased phosphatidylcholine and phosphatidylethanolamine levels, which improved metabolic process [21]. In the pancreatic applications, Melamed et al. found that LNP-encapsulated mRNA containing the cationic lipid DOTAP targets pancreatic cells [47]. In the gastrointestinal tract, Sung et al. reverse-engineered an LNP system, mimicking ginger nanoparticles to deliver the lipid composition of ginger nanoparticles, enabling oral delivery to colonic mucosa, increased expression of IL-22 protein, and accelerated epithelial repair [22]. Li enhanced mucosal immune responses by co-delivering mRNA vaccines and ATRA via LNPs [48], and Suri et al. alleviated colonic inflammation by delivering miRNA-146a mimics [49]. All these studies highlight the advantages of LNPs for the targeted delivery of various nucleic acid drugs.

LNPs have achieved notable advancements in clinical applications, particularly in cancer treatment [50] (Table 3). For instance, the NAPOLI-1 study highlighted the survival benefits of combining liposomal irinotecan with 5-FU/LV for patients with metastatic pancreatic cancer who had not responded to gemcitabine treatment. This combination increased median overall survival from 4.2 to 6.1 months (HR 0.67) [51]. In the NAPOLI 3 trial, the NALIRIFOX regimen (liposomal irinotecan, oxaliplatin, and 5-FU/LV) served as a first-line treatment for metastatic pancreatic cancer. Compared to gemcitabine plus albumin-paclitaxel, it extended median overall survival to 11.1 months (HR 0.83) [52]. Additionally, the NIFTY trial confirmed that liposomal irinotecan combined with 5-FU/LV significantly improved progression-free survival in second-line treatment for advanced biliary tract cancer, with median PFS increasing from 1.7 to 4.2 months [53]. In the realm of patents, liposomal irinotecan, marketed as Onivyde, was developed by Merrimack Pharmaceuticals and received FDA approval. Meanwhile, HR070803, developed by Hengrui Medicine, gained approval in China based on the PAN-HEROIC-1 study results [54]. These examples illustrate the comprehensive journey of LNPs from preclinical research to commercial application.

LNPs have potential for delivering drugs, but are a bit tricky to administer. Intravenous injection allows systemic delivery, but accumulates in non-target organs, possibly leading to off-target effects. Melamed noted that cationic lipid LNPs are strongly distributed in other organs than the pancreas (e.g., the spleen), which may lead to non-specific immune reactions [47]. In contrast, oral LNPs face challenges such as degradation by enzymes, mucus barriers, and acidic gastrointestinal environments. Sung has improved stability by biomimetic lipid design; however, the encapsulation rate and intestinal absorption efficiency of oral LNPs are much lower than those of injections [22]. Endoscopic techniques with direct access to the gastrointestinal tract have been used for injection and spray. Applying these techniques to LNPs is a promising strategy that allows delivery of nucleic acids directly to the lesion mucosa, bypassing the intestinal epithelial barrier, gastrointestinal degrading enzymes, and thus enhancing local bioavailability.

#### 2.1.2. Biodegradable Polymer Nanoparticles: Sustainable Local Chemotherapy

Polymer nanoparticles are increasingly preferred as drug delivery carriers due to their customizability, biocompatibility, and degradability. Among them, polylactic-co-glycolic acid (PLGA) is the most popular one [58]. PLGA is a synthetic polymer formed by ring-opening polymerization of LA and glycolic acid, degradation products are naturally occurring in human metabolic pathways and provide high biological safety [59]. The US FDA has approved PLGA for various medical devices and controlled release formulations, underscoring its clinical viability due to its biocompatibility and degradability [60,61]. Its degradation rate and drug-release kinetics can be tuned by changing the lactic acid/glycolic acid ratio and the molecular weight, allowing drug release to occur over days or months [62]. PLGA nanoparticles are versatile and can be used to encapsulate hydrophobic drugs, hydrophilic drugs, macromolecules like proteins, and nucleic acids. PLGA nanoparticles can also be functionalized and chemically modified using targeted ligands (folic acid, transferrin, antibody) or stealth coating [59].

In the treatment of gastrointestinal disease, PLGA nanoparticles are promising drug delivery platforms. Azevedo et al. developed surface-engineered albumin PLGA-PEG nanoparticles to enhance oral insulin absorption through FcRn receptor transport and improve blood glucose control in diabetic models [63]. Diniz et al. used PLGA nanoparticles targeting Sialyl-Tn antigen to deliver Foretinib successfully overcoming tumor resistance and preventing metastasis in gastric cancer [23], and Xiao showed that Icariin-loaded PLGA nanoparticles improve antitumor effects by reducing mitochondrial oxidation [24]. Alam et al. showed that PLGA nanoparticles delivering curcumin exhibit stronger antibacterial activity against Helicobacter pylori and induce apoptosis in gastric cancer cells by modulating Bax/Bcl-2 ratio [64], and Gao et al. used cancer cell membrane coating technology to construct biomimetic nanoparticles for co-delivering doxorubicin and curcumin, significantly improving targeting and reducing cardiotoxicity in esophageal cancer models [25]. Bao et al. co-loaded Erlotinib and Daphnetin into PLGA nanoparticles, offering a new approach for pancreatic cancer treatment [65]. Collectively, these studies show that PLGA nanoparticles provide different solutions for gastrointestinal disease treatment through targeted delivery, immune modulating, and multi-pathway synergy.

Other than PLGA particles, polysaccharide and Eudragit^®^ S100 (ES100) nanoparticles are other types of polymer nanoparticles [66]. Polysaccharide nanoparticles exploit the biocompatibility and degradation of natural polysaccharides such as chitosan, sodium alginate, and glucan. They are synthesized electrostatically, covalently cross-linked with ion-gel, and used to deliver oral protein/peptide drugs to improve intestinal barrier penetration and targeting. Chitosaninsulin nanoparticles improve oral bioavailability by improving mucus adhesion and tight junctions [67]. ES100 nanoparticles, a pH-sensitive methacrylic acid copolymer, dissolve in a high pH colon (pH > 7.0), and allow colon-specific drug release [68]. For example, ES100-coated PLGA nanoparticles have been shown to effectively deliver etoricoxib for treating inflammatory bowel disease [69]. Similarly, eluxadolin-loaded Eudragit nanoparticles (ENPs) have proven potential for treating irritable bowel syndrome diarrhea (IBS-D) by enhancing drug stability and targeting, thereby improving therapeutic efficacy [70]. Furthermore, polysaccharides and ES100 nanoparticles hold significant promise for the oral delivery of protein/peptide drugs and for colon-targeted therapies.

Similar to LNPs, polymer nanoparticles encounter difficulties in treating pancreatic cancer due to the dense extracellular matrix, poor blood supply, difficulty of systemic chemotherapy drugs in reaching tumors, and drug resistance. Using the well-known EUS-guided fine-needle aspiration technique in digestive endoscopy [71,72,73], direct injection of PLGAs loaded with chemotherapy drugs into the tumor may result in a high concentration at the tumor site, keeping the concentration high for longer, and targeting and killing tumor cells while minimizing severe systemic side effects like bone marrow suppression.

#### 2.1.3. Inorganic Nanoparticles: A Cutting-Edge Platform for Integrated Diagnosis and Treatment

Inorganic nanoparticles made from gold, iron oxides, and silicon are nanocarrier systems with controllable physical properties, unique optical, magnetic, or radioactive properties, easy surface functionalization, and easy surface functionalization. These properties have been widely studied [38]. They offer the advantage of integrating diagnostic imaging and treatment simultaneously.

Mesoporeous silica nanoparticles have a high specific surface area and pore volume that allow efficient loading of drug molecules. Their surfaces can easily be functionalized, attaching to targeted molecules or stimulus-responsive caps for controlled release [74]. In gastrointestinal applications, MSNs loaded with chemotherapy drugs are used for targeted colorectal cancer treatment and can be modified to release drugs responsively in the tumor microenvironment. Narayan et al. used modified MSNs modified with a chitosan-glucuronic acid copolymer as a capecitabine carrier [75].

AuNPs are highly effective CT contrast agents because of their large number, surface plasmon resonance effect, and suitability for photothermal therapy (PTT) and photodynamic therapy (PDT) [76]. Researchers developed a multifunctional gold nanocage platform for diagnosis and treatment. Using photothermal conversion power, gold nanocages can target tumor cells and release antigens, and also deliver immune drugs to improve the tumor microenvironment. This technique inhibited both primary and metastatic tumors in animals and is a promising strategy for metastatic gastroenterology [26].

Iron oxide nanoparticles (IONPs) are superparamagnetic nuclear magnetic resonance (MRI) contrast agents. They can help to localize lesions before treatment and evaluate efficacy after treatment, and produce heat if exposed to alternating magnetic fields, making them suitable for magnetic hyperthermia and drug carriers [77]. Li et al. have proposed a multifunctional diagnostic and therapeutic PTT with enzyme-catalyzed enhanced ferroptosis, which is a potent tumor cell-killing mechanism both in vivo and in vitro. The Fe_3_O_4_ core enhances MRI capabilities, which is a potential diagnostic tool [27].

Inorganic nanoparticles are highly versatile and are ideal for precise endoscopic treatment. Gold nanoparticles are widely used as multifunctional nanocatalysts and contrast agents. They enhance imaging contrast, trigger photothermal therapy, and control drug release, all under endoscopic systems like endoscopy and confocal endoscopy, thereby improving cancer diagnosis and treatment [78]. MSNs loaded with fluorescent dyes and chemotherapy drugs can precisely resect tumors using endoscopic fluorescence imaging. Postoperatively, they can release drugs to remove residual tumors. In addition, iron oxide nanoparticles can be integrated with endoscopic technology, providing a platform for precise positioning, local synergy treatment, and real-time imaging monitoring, and thus offer a promising approach for minimally invasive general treatment of gastrointestinal tumors.

#### 2.1.4. Discussion on the Physicochemical Compatibility of Endoscopic Injection Systems with Nanoparticles

As previously noted, the digestive endoscopic injection system can significantly enhance the delivery efficacy of nanoparticles. In this nanoparticle delivery system, the key performance parameters of the endoscopic injection pipeline and needle must be meticulously aligned with the physicochemical properties of the nanomedicine to optimize both delivery efficiency and therapeutic outcomes.

Size compatibility is the foremost consideration. The inner diameter of the pipeline and the gauge size of the needle should be tailored to the particle size and aggregation tendencies of the nanoparticles. For instance, smaller nanoparticles, such as liposomes measuring less than 100 nm, can be accurately injected using fine-bore needles, such as 27G. Conversely, larger or high-viscosity nanosuspensions, such as polymer microspheres, necessitate pipelines with larger inner diameters to prevent clogging and structural damage due to shear forces [38]. Furthermore, the bevel design of the needle and the geometry of the opening, which may include multi-sided holes or conical tips, influence the uniformity of nanoparticle release and the depth of tissue penetration. Therefore, these features must be aligned with the diffusion characteristics of the nanoformulations [79].

Material compatibility is of paramount importance. The materials used in pipes and needles, including medical stainless steel, polytetrafluoroethylene, and silicone coatings, must be biologically inert to avoid chemical interactions or adsorption with nanoparticle surfaces. For instance, cationic lipid nanoparticles are prone to binding with negatively charged metal surfaces, which diminishes delivery efficiency. Consequently, it is essential to employ neutral coatings or modified materials [80]. Furthermore, the mechanical properties of these materials, such as flexibility and wear resistance, must accommodate the bending paths encountered during endoscopic procedures while ensuring the stability of nanoformulations.

In terms of design, the geometry of the pipeline—whether straight, retractable, or featuring integrated imaging functions—must align with the working channel of the endoscope to maintain controllability in complex anatomical environments. The length and rigidity of the needle must strike a balance between tissue penetration and operational accuracy. For example, a longer needle may be advantageous for deep tumor injections but could increase the risk of deviation, whereas shorter needles are more appropriate for submucosal injections [78]. Additionally, some advanced designs incorporate real-time monitoring capabilities, such as fluorescence or ultrasound guidance, to fulfill the tracking requirements of nanoparticles, including surface-modified ligand-targeted particles.

The physicochemical properties of nano-preparations, including surface charge, hydrophilicity, hydrophobicity, and stability, necessitate that the injection system possesses corresponding adaptability. For example, PEG-modified nanoparticles can minimize pipe adhesion; however, it is essential to avoid compatibility issues with specific polymer materials. In the case of temperature-sensitive nanomaterials, such as heat-responsive liposomes, the pipeline material must exhibit either heat-insulating or heat-conducting properties to preserve functional integrity [81]. Additionally, the concentration and viscosity of nanoparticles influence flow dynamics, necessitating the optimization of delivery parameters through adjustments to the smoothness of the inner wall of the pipe or the inner diameter of the needle.

In conclusion, the endoscopic injection system can achieve physicochemical compatibility with nanoparticles, but it requires the joint optimization and adaptation of the endoscopic injection system and nanoparticles to jointly achieve more precise drug delivery.

### 2.2. In Situ-Formed Hydrogels: Smart Materials for Wound Management and Drug Storage

In situ-formed hydrogels are “intelligent” biomaterials with unique phase transition behavior, which can be used for drug delivery for limited intracavitary applications [82]. They are usually liquid sol particles outside the body, easy to transport through small-sized instruments. When they touch target tissues (temperatures, pH, or strengths), they undergo rapid physical or chemical cross-linking to become hydrated gels with a 3D network structure [83,84,85,86]. This transition from solution to gel allows them to adhere easily to irregularly shaped tissues, form uniform protective coatings, and overcome problems of traditional preformed implants, such as imprecise implantation and poor adhesion [87,88]. As drug carriers, hydrogel porous structures can capture different drug molecules. By changing their cross-linking density or degradation rate, drug release kinetics can be controlled, from several days to weeks, to maintain a stable local drug concentration [88,89].

In situ-formed hydrogels have been studied for various clinical problems. Hydrogels that can change in situ and adhere to irregular wounds can act as “smart bandages” for the digestive tract. Lei and colleagues developed a dual-network in situ hydrogel made of sodium alginate and gelatin. The hydrogel provides a mechanically adaptive barrier against calcium ions by calcium cross-linking, and protects wounds after ESD and promotes mucosal healing [90]. Xu and colleagues developed a pH-responsive hydrogel (Cur/SA/PC) with sodium alginate (SA) and polyaspartic acid stabilized CaCO3 (PC) to load curcumin (Cur) gels in gastric acid, creating a barrier that promotes Cur bioavailability [91]. The porous hydrogels are also used for drug release. Siripruekpong and colleagues designed a gastric retention raft system with low/medium viscosity sodium alginate, pectin, or gellan gum coloading solid dispersions of curcumin and resveratrol, which forms a floating gel raft in the gastric juice, allowing continuous drug release (>80% over 8 h) and cytotoxicity and anti-inflammatory effects against human gastric adenocarcinoma cells [92]. Jiang et al. developed a thermosensitive hydrogel loaded with OXL liposome (OXL/IL-15 TG) and a thermosensitive hydrogel loaded with OXL/IL-15 TG, which provides a drug reservoir targeted tumor delivery of oxaliplatin and maintains IL-15 release [30]. The aforementioned cases indicate that in situ-formed hydrogels exhibit great potential in the treatment of gastrointestinal diseases due to their injectability, environmental responsiveness (e.g., to pH and temperature), and bioadhesiveness.

The combination of in situ-formed hydrogels and digestive endoscopy technologies is a very promising example of precision medicine in the treatment of gastrointestinal disorders. Endoscopy is a minimally invasive approach to the disease, and hydrogels extend the therapeutic potential of endoscopy beyond diagnosis and resection. For example, after ESD or EMR, physicians can apply hydrogels with hemostatic drugs or growth factors on the ulcer surface using spray catheters. The in situ “smart bandage” isolates the wound and releases drugs over time to help mucosal healing and reduce the need for titanium clips. Zhou et al. developed PCAC16 hydrogel with charge-reversal antibacterial molecules, which was applied to ulcer surfaces post-ESD in pig stomachs via endoscopy. They demonstrated that it targets Helicobacter pylori and promotes ulcer healing [29]. Similarly, via endoscopy, the colloid solution (Konjac Glucomannan + Sodium Alginate) and the fixing solution (ε-Polylysine + Calcium Chloride) can be sequentially sprayed onto the ESD-induced wounds in the esophagus and colon of pigs. A hydrogel is then formed in vivo, which can promote wound healing and inhibit fibrosis [32]. In cases of gastric perforation, Ni et al. reported that PPCL@Mg dry powder was sprayed onto the gastric perforation site of pigs using an electric endoscopic powder delivery device. The powder spontaneously formed a hydrogel after absorbing interfacial water, achieving suture-free closure. This procedure was successfully performed in pig models, and its wound-healing promotion effect was confirmed [31]. Liu et al. introduced a hyperbolic hydrogel device to a perforation site in the pig stomach by endoscopic forceps. The device expanded in the acidic gastric environment to seal the perforation. pH changes are monitored by a capsule robot [93].

Finally, in situ-formed hydrogels can be used for localized treatment of gastroenterology since they provide in situ formability and controlled drug release properties. When combined with gastrointestinal endoscopy techniques, they improve therapeutic results and help endoscopic treatment from mechanical treatment to precise and sustainable drug treatment.

### 2.3. Drug-Eluting and Biodegradable Stents: Long-Term Treatment of the Gastric Lumen

Gastrointestinal lumen stenosis, caused by malignant disease or due to benign disease, is a major clinical challenge in gastroenterology. Self-expanding metal stents are widely used to treat obstructions and improve patient quality of life [94,95]. However, the limitations of traditional stents have become increasingly prominent, such as re-obstruction caused by tumor regrowth and the long-term risks of permanent implants in benign diseases [96,97,98]. Drug-eluting stents and biodegradable stents have been developed to overcome these limitations. They provide more than mechanical support: they are endovascular treatment platforms for localized and continuous delivery of drugs directly at the lesion site.

#### 2.3.1. Drug-Eluting Stents

Drug-eluting stents provide a reliable mechanical support while actively treating disease by local drug delivery. The key ingredient of the design is a drug coating on the surface of the stent. Typically, a biocompatible polymer matrix is applied over the metal stent, where therapeutic drugs are embedded [35]. When the stent expands at the restricted point, the drug is released in a controlled manner. The result is a high concentration of therapeutic drug in the immediate area and lower systemic toxicity and side effects [33,99]. The main advantage of this local administration is precise targeting.

Combining drug-eluting stents with digestive endoscopy systems gives the benefit of complete treatment in one procedure. The precise release of stents guided by endoscopic direct vision or ultrasound allows for covering the lesion segment accurately with both the stent and drug effects. This combination of immediate intervention of endoscopy (i.e., reducing obstructions) and the continued drug delivery function of stents makes the “implacement as treatment” model possible. Drug-eluting stents (DESs) are promising for digestive endoscopy interventional therapy. They provide mechanical support and local drug delivery. Their main advantage is that they provide complete treatment in one intervention. Under direct endoscopy or ultrasound guidance, these stents precisely release drugs targeted to the lesion. Combining the immediate obstruction relief capability of endoscopy with the sustained drug treatment function of a stent creates an efficient “implacement as treatment” model. Various research teams have developed DESs for digestive tract diseases. For example, Jang et al. developed a paclitaxel-eluting metal stent (MSCPM-III) for malignant biliary obstruction. It contains a polytetrafluoroethylene (PTFE) membrane with sodium octanate to increase drug penetration. A multicenter randomized controlled trial showed that the stent did not extend patency time but reduced tumor volume in cholangiocarcinoma patients and showed local anti-tumor effects [35]. In esophageal cancer treatment, Fouladian et al. developed a docetaxel-eluting polyurethane-coated nickel–titanium alloy esophageal stent using the dip coating method. In vitro studies showed that this stent can continuously release docetaxel for more than 30 days effectively induce apoptosis in KYSE-30 cells (KYSE-30 cells) with anti-cancer activity comparable to pure drugs [100]. In gastrointestinal cancer-induced obstructions, Arafat et al. reported a 5-fluorouracil (5FU) eluting stent that consists of a double-layer coating (polyurethane base and polyethylene-vinyl acetate cover layer) for continuous 5FU for 161 days. This product meets regulatory quality requirements [99]. In addition, regarding the challenging condition of chronic pancreatitis complicated with pancreatic duct stones, Li et al. designed hydrogel-coated pancreatic stents loaded with citric acid (CA), using gelatin methacryloyl (GelMA) and carboxymethyl chitosan methacryloyl (CMCSMA) as the matrices, respectively. In vitro experiments have demonstrated that these stents can release citric acid efficiently and in a controlled manner, dissolve over 90% of pancreatic duct stones within 3 days, and simultaneously exhibit excellent biocompatibility—thus providing an innovative drug-eluting solution for the endoscopic treatment of pancreatic duct stones [33,101]. Collectively, these cases indicate that DESs, by locally delivering chemotherapeutic drugs (such as paclitaxel, docetaxel, and 5-fluorouracil) or chemical dissolvents (such as citric acid), provide a more precise, efficient, and safe treatment option for addressing challenging issues in the field of digestive endoscopy, including the treatment of malignant obstruction and the dissolution of stones.

#### 2.3.2. Biodegradable Stents

Biodegradable scaffolds are a new design approach that is primarily temporary. These scaffolds consist of high-molecular-weight materials, such as polylactic acid and polycaprolactone, that are hydrolyzed and metabolized in the body [37]. First, they provide mechanical support after implanting, then, as the narrow area shrinks and stabilizes or the anastomosis heals, they slowly degrade into harmless byproducts that the body absorbs, minimizing long-term risks of permanent implants and eliminating the need for secondary surgery to remove them [34]. This characteristic endows it with unique advantages in the treatment of benign digestive tract stenosis.

Integrating digestive endoscopy with biodegradable stents allows for precise and controllable placement at lesion sites with temporary support. This is a paradigm shift from “permanent implantation” to “temporary support and treatment”, particularly for young patients with growth needs or benign conditions. Lee et al. demonstrated the role of endoscopic methods for implanting degradable stents. They successfully used endoscopic retrograde cholangiopancreatography (ERCP) and direct transoral choledochoscopy (DPOC) to implant a dexamethasone-loaded sheath/core structure degradable biliary stent in a pig model with benign BBS. The stent maintains biliary patency, expands narrow areas, and promotes ulcer healing in 12 weeks, completely degrading by 16 weeks in order to avoid removal surgery [34]. Liu et al. developed a magnesium-based braided stent coated with PLGA and supported with paclitaxel (PTX) in a rabbit model of corrosive esophageal stenosis. Follow-up endoscopy and esophageal biopsy revealed that the stent maintained the esophageal lumen patency for 3 weeks and reduced inflammation and fibrosis, showing that endoscopy and degradable stents may be useful for benign esophageal stenosis treatment [102]. In terms of material technology innovation, Zhao et al. proposed a unidirectional biodegradable stent system based on a single poly-L-lactic acid (PLLA) material. This advancement in materials science has greatly enhanced the flexibility of endoscopists in selecting instruments when treating biliary tract diseases, providing a new tool for personalized endoscopic treatment [36]. Collectively, these cases indicate that the combination of biodegradable stents and digestive endoscopy technology has become a minimally invasive, effective, and highly promising method for treating gastrointestinal stenotic diseases.

In conclusion, drug-eluting stents and biodegradable stents have broadened the scope of endovascular treatment through distinct approaches. Drug-eluting stents improve the management of malignant diseases by enabling “continuous administration.” In contrast, biodegradable stents offer a more practical solution for benign conditions by providing “temporary support.”

## 3. Advanced Strategies for Enhancing Treatment Accuracy

While the bioengineering platform mentioned above has delivered local drugs, therapeutic results must be targeted to specific cells and released promptly. Two strategies for this aim have been developed (active targeting and stimulus-responsive release). Both are designed to achieve a “dual control of time and space” in precision medicine. They act as a navigation system and an intelligent switch to deliver drugs [38,103,104].

### 3.1. Active Targeting

The active targeting strategy, which provides delivery systems with “navigation”, is achieved by covalently changing carriers like nanoparticles and liposomes’ surfaces by binding specific ligands to receptors recognizing overexpressed markers on diseased cells’ surfaces. This means that the drug carrier can be directed directly to target cells [105]. This increases the concentration of vector at the target and keeps the vector in target cells, eliminating the physiological barrier and minimizing off-target effects on healthy tissue.

Several studies have shown that disease-specific drugs can be targeted through ligand–receptor interactions. For pancreatic cancer, a PEG-modified gold nanoparticle (ACG44P1000) uses Cetuximab as a targeting agent and Gemcitabine as the chemotherapy payload to actively target EGFR co-expressed on pancreatic cancer and stellate cells for precise treatment of pancreatic ductal adenocarcinoma (PDAC) [28] For gastrointestinal cancer cells overexpressing specific membrane proteins, high affinity nucleic acid aptamers (aptamers targeting mucin 1) recognize MUC1 on colorectal cancer cells to construct targeted delivery systems and provide novel treatment for colorectal cancer [106]. In addition, Salmonella vectors (VNP20009) are used as delivery platforms to transport aptamer-conjugated drugs (APdc) to pancreatic tumors using their natural tumor chemotaxis and paving the way for biological vectors in targeted solid tumor therapy [107]. Natural affinity of lectins for colonic mucosa can be used to target inflammation and tumors [107]. Together, these examples highlight the precise application value of active targeting strategies.

Endoscopic technique local drug delivery reduces the non-specific drug distribution. By incorporating active targeting vectors, “re-targeting on a local basis” is possible. Targeted drug-loaded nanoparticles can be sprayed on early cancerous lesions under direct endoscopic vision. They can concentrate in cancerous epithelium. This can increase the treatment of residual lesions or marginal areas and thus lower the recurrence rates after traditional endoscopic resection.

### 3.2. Stimulus-Responsive Release: Installing a Controllable “Smart Switch”

The stimulus-responsive strategy is to create a system that detects the microenvironment of the lesion or external stimulus signals and triggers the drug release. This allows precise control over the release time, allowing “on demand drug administration” [108]. These systems are generally classified into two types: endogenous stimulus responses using the internal environment and exogenous stimulus responses activated by external devices.

Endogenous stimulus responses exploit the differences in pH between lesion and normal tissues. For example, the pH response exploits the changes in the pH gradients between the stomach (pH 1.5–3.5) and colon (pH 7.0), designed acid-unstable bonds, such as silicone–ether bonds, to protect drugs like gemcitabine (GEM) and release active agents under specific intestinal pH conditions [109]. Drug delivery systems include hydrogels. Jiang et al. developed a pH-responsive amphoteric ionic hydrogel made from bacteria and chitosan, which has the lowest swelling rate at pH 3.5–5.0 but increases in acidic or basic environments. Drug release is low (37.5%) in simulated gastric juice (pH 1.2), and increases (up to 77.5%) in simulated intestinal fluid (pH 6.8/7.4), and is therefore suitable for intestinal drug delivery [109]. Moreover, many tumors and inflammation sites overexpress certain enzymes, such as matrix metalloproteinases or cathepsin. Enzyme-triggered release can be obtained by linking drugs to carriers with peptide chains cleavable by these enzymes. Researchers have used cathepsin B expressed in cell lysosomes to cleave peptide chains such as GFLG, triggering the release of prodrugs such as docetaxel (DTX). While used for tumor treatment, the enzyme-triggering strategy can be used for gastrointestinal mucosal disease treatment [110]. Response systems based on endogenous small molecules also show promise. ATP-responsive mesoporous silica nanoparticles selectively release drugs in tumor intracellular environments with high ATP by functionalizing ATP aptamers [111].

Exogenous stimulus responses provide precise spatiotemporal control, which is ideal for integration with endoscopic treatment platforms. Near-infrared light, known for its effective penetration of tissue, was used by researchers using platinum (IV) prodrugs with nanoparticles. It enables targeted release of chemotherapy drugs at tumor sites under near-infrared light, protecting normal tissues [112]. Schoellhammer et al. developed an RNA delivery system using ultrasound stimulation. They enhanced the colon’s mucosal permeability to macromolecules by using a transient spatiotemporal effect from low-frequency ultrasound (20–40 kHz), and delivered TNF-specific siRNA to a mouse model of DSS-induced colitis [113]. These stimulus-response strategies allow precise drug release at specific times and locations using external energy applied to the intestinal tract. This greatly increases therapeutic effects and minimizes systemic side effects. A digestive endoscopy system has potential as a transmitter of two types of external stimuli. With the existing digestive endoscopy photodynamic therapy, emitting near-infrared light through the endoscopic channel is feasible. Endoscopic ultrasound operates at a different frequency than low-frequency ultrasound used in ultrasonic stimulation. However, it is technically possible to deliver a miniaturized low-frequency probe through the endoscopic channel.

Finally, active targeting and stimulus-responsive release can often be combined in one “intelligent” delivery platform. For example, a nanoparticle with a targeting ligand and pH-responsiveness can target tumors with active targeting and release drugs as soon as the tumor is slightly acidic, for precise cascade delivery. Furthermore, the digestive endoscopy system can deliver exact doses and spatiotemporal accuracy of external stimuli. Combining multiple platforms will make the treatment of gastrointestinal disease more efficient, safer, and controllable.

## 4. Translational Medicine Perspective: Challenges from Laboratory to Hospital Bed

Bioengineered drug delivery platforms for digestive endoscopy, while promising in preclinical studies, encounter significant challenges in moving from the lab to everyday clinical use. Successfully navigating this transition demands the concerted efforts of researchers, clinicians, regulatory bodies, and industry professionals to tackle several critical issues collaboratively.

### 4.1. Biosafety and Long-Term Compatibility: Surpassing Acute Toxicity Assessment

The most important issue is ensuring biological safety. The digestive tract is a complex environment with unique microbial communities, enzyme systems, and physicochemical gradients (e.g., pH), and the long-term effects and fate of new materials such as nanoparticles and hydrogels are unknown. For example, biodegradability and clearance pathways of long-retained inorganic nanoparticles must be defined to avoid accumulation of toxicity. These nanoparticles are often poorly biocompatible and may exhibit unexpected toxicity due to their metallic properties [114,115]. Other common crosslinking agents in hydrogel preparation (e.g., EDC and glutaraldehyde) provide necessary physical properties but may cause irritation or toxicity. It is important to control and remove remaining amounts of these agents before applying them in vivo [116].

Endoscopic local injection, compared to traditional intravenous administration, typically results in higher drug concentrations at the target site. However, if not controlled, these concentrations can cause severe local tissue toxicity, including inflammatory responses, necrosis, and even perforation. For instance, the injection of TNFerade, an adenovirus vector expressing TNF-α, led to local inflammatory responses such as pancreatitis and cholangitis, which were strongly associated with the dosage and local drug buildup [117]. Additionally, excessive local drug concentrations may trigger systemic toxicity, such as fever or cytokine storms due to immune activation, particularly noted with the injection of immunomodulators like dendritic cells [118].

Future strategies should prioritize precise dose control and the design of intelligent carriers. Optimizing injection techniques and incorporating real-time imaging methods, such as EUS or fluorescence-guided approaches, are crucial for monitoring drug distribution. This allows for dynamic adjustment of injection strategies to prevent single-point drug accumulation [81]. Furthermore, individualized dose calculations based on tumor volume, vascular density, and patient-specific metabolic differences are vital for minimizing toxicity risks [119]. Beyond current cytotoxicity tests and short-term animal studies, comprehensive toxicological research supported by extensive experimental data is necessary. This approach ensures that materials exhibit low toxicity and minimal immunorejection, thereby enhancing patient safety and treatment efficacy.

### 4.2. Large-Scale Production and Robust Quality Control: The Gap from Milligrams to Kilograms

In the lab, researchers can easily produce small quantities of nanoformulations of optimal size and high drug loading. Scaling up for clinical trials and commercialization, e.g., GMP standards, batch-to-batch consistency can be a challenging engineering task [120]. During scale-up, important nanoparticle parameters such as particle size distribution, surface charge, drug encapsulation rate, and release velocity can vary. Even small deviations in such parameters can impact in vivo behavior and efficacy. Size change alone can have a major impact on target, off-target toxicity, and drug release [39]. In vivo hydrogels utilized for endoscopic injection require precise control over gelation temperature, viscosity, and strength. Deviations in these parameters can result in blockage of the endoscopic working channel or hinder normal gelation in vivo. Consequently, establishing a stable, repeatable, and economically viable large-scale production process is essential for these platforms to successfully penetrate the market.

To address the challenges associated with large-scale production, it is essential to implement the concept of “Quality by Design” (QbD) [121]. This approach necessitates a comprehensive understanding and definition of key material attributes (CMAs) and key process parameters (CPPs) during the early stages of research and development, thereby establishing a reliable design space [4]. Utilizing process analytical technologies (PAT), such as online dynamic light scattering (DLS) or Raman spectroscopy, enables real-time monitoring and feedback control of critical parameters, including particle size and the polydispersity index (PDI). This capability ensures the robustness of the production process [80]. Continuous manufacturing and microfluidic synthesis technologies represent promising avenues for enhancing production efficiency. These methods facilitate more precise mixing and reaction control, which can lead to increased yields, reduced batch-to-batch variability, and lower production costs [4,121]. In the future, modular production line designs are expected to better accommodate the small-batch and multi-variety production needs associated with personalized nanomedicines, such as individualized cancer vaccines [80]. Concurrently, the development of low-cost, biodegradable “green” synthetic materials, including specific formulations of PLGA and lipids, along with the simplification of formulation design—such as minimizing unnecessary target head modifications—will help control costs from the outset [4].

### 4.3. Complex Regulatory Approval Path: Navigating the Maze of Drug-Device Combination Products

The combination of bioengineering delivery platforms and endoscopic technology produces a “drug-device combination product,” where both drugs and devices must be approved according to regulatory criteria, making them more complicated than single products [122]. Regulatory bodies evaluate product safety and efficacy, but must explicitly define whether the product is device-led or drug-led, which directly affects the design and strategy of clinical trials. For example, when a PLGA nanoparticle loaded with chemotherapy drugs is delivered via a standard endoscopic needle, the nanoparticle is the drug, while the needle is the device. On the other hand, if a special endoscopic accessory with an integrated microneedle array is developed, the entire system may be classified as a new type of device. This classification poses significant regulatory risks and time costs to developers.

Developers must engage with regulatory authorities early and often to foster international collaboration. Establishing a platform akin to the EU REFINE project can enhance data sharing and standardization among academia, industry, and regulatory bodies [38]. This approach aims to identify the most efficient approval path.

### 4.4. Clinical Feasibility and Cost-Effectiveness: Demonstrating Its Clinical and Economic Value

The success of any new technology in healthcare depends on its ability to provide clear value to patients and the medical system. Clinically, a new delivery platform must fit seamlessly into existing endoscopic workflows without significantly increasing the operating time or complexity. For example, a nano-suspension requiring complex pre-operation preparations or special equipment may see lower clinical adoption than standard solutions. Health Economics Analysis: What cost–benefit advantage does it have over standard therapies? Local targeted drug delivery may reduce systemic side effects and treatment costs, but high costs of research, development, and production might not account for these savings. For medical insurance payers, it is vital to show that the new technology significantly improves key clinical endpoints, such as overall survival and quality of life, rather than improving other metrics like bioavailability or drug concentration in tumors. In summary, it is not easy to achieve these transformation challenges without collaboration from a multidisciplinary team. Better communication between basic research and clinical needs, taking into account large-scale production and regulation early in the development process, will be crucial to deliver these promising platforms to patients.

Enhancing cost-effectiveness necessitates a multi-faceted approach [123]. Initially, production costs can be minimized through technological advancements. For example, adopting continuous flow production processes can boost yield, while developing universal and modular nanoplatforms, like universal LNP platforms capable of carrying various drugs, can distribute initial R&D expenses [38]. Additionally, optimizing the supply chain and promoting large-scale, standardized production of essential excipients, such as ionizable lipids, can lower procurement costs [124]. In clinical development, focus should be directed towards areas with significant unmet medical needs where nanotechnology offers substantial benefits, such as genetic disorders and refractory tumors. Carefully structured clinical trials are essential to demonstrate that these technologies provide notable cost-effectiveness advantages over traditional therapies [1,2]. Looking ahead, gathering long-term efficacy, safety, and economic data on new drug delivery systems through real-world evidence (RWE) will be vital for medical insurance negotiations and pricing strategies [124]. Simultaneously, exploring innovative business models, like risk-sharing agreements based on treatment outcomes, can mitigate initial risks for payers and enhance market access for new drug delivery systems [123].

## 5. Conclusions and Future Prospects

In conclusion, this review has articulated the transformative potential of integrating bioengineered drug delivery platforms—nanoparticles, in situ hydrogels, and advanced stents—with therapeutic gastrointestinal endoscopy. This synergy transcends the traditional diagnostic and resectional roles of endoscopy, enabling precise, localized, and sustained therapy that directly addresses the limitations of systemic drug administration.

A critical appraisal of the current landscape, however, reveals that many studies remain at the proof-of-concept stage, often demonstrating technical feasibility in isolation. A significant gap exists between engineering novelty and clinical practicality. True progress will depend not merely on developing new materials but on a deeper, more collaborative focus on platform-clinical workflow integration. This includes designing systems specifically for endoscopic delivery (e.g., suitable viscosity for spraying, stability during passage through the endoscope channel) and conducting robust preclinical studies in disease-relevant models that mimic human pathophysiology.

Looking forward, we propose several key perspectives that should guide future research to realize the full potential of this field. Hyper-personalization: Future platforms will evolve from “one-size-fits-all” to being tailored using patient-specific data, including genomic profiles of tumors, the local microbiome, and individual healing responses [80,125].

Intelligent Systems powered by AI and Robotics: The integration of artificial intelligence for real-time analysis of endoscopic images to precisely delineate lesions and guide automated, targeted drug delivery will be crucial [126,127,128,129]. Furthermore, robotic-assisted endoscopy could enhance the stability and precision of platform deployment.

Convergence of Diagnosis and Therapy (True Theranostics): The next generation of platforms will be inherently theranostic, capable of simultaneous lesion characterization (e.g., via integrated contrast agents), targeted drug delivery, and real-time monitoring of therapeutic efficacy, all under endoscopic guidance [130,131].

From our perspective, the journey ahead is as much about engineering and medicine as it is about fostering a new culture of collaboration. The most significant breakthroughs will likely emerge from deeply integrated teams where bioengineers, clinicians, and regulatory scientists work side-by-side from the inception of an idea. While substantial challenges in scalability, regulatory approval, and cost-effectiveness remain, the potential to shift the paradigm of GI disease management from repetitive, systemic treatments to definitive, minimally invasive, and localized interventions is undeniable. We believe that by addressing the outlined challenges and pursuing the proposed directions, this interdisciplinary approach is poised to revolutionize therapeutic endoscopy, ultimately delivering on the promise of precision medicine for gastrointestinal patients.

## Figures and Tables

**Table 1:** Comparative analysis of major bioengineering platforms for endoscopic drug delivery.

Platform Category	Core Materials	Key Advantages	Primary Challenges	Endoscopic Application Scenarios
Nanoparticulate Systems (e.g., LNPs, polymeric NPs, inorganic NPs)	Ionizable lipids, PLGA, gold/silica/iron oxide NPs, et al.	Cellular/Subcellular precision, theranostic capabilities, high drug loading capacity	Long-term biosafety, scalability, and quality control, targeting efficiency	EUS-guided intratumoral injection, targeted spraying on early-stage lesions, molecular imaging, et al.
In Situ-Forming Hydrogels	Alginate, chitosan, thermoresponsive/pH-responsive polymers, et al.	Conformable coverage of irregular wounds, sustained release (days to weeks), ease of endoscopic delivery	In vivo stability and retention, precise control of degradation kinetics	Post-ESD/EMR “smart bandages”, gastric perforation sealing, Local drug depots, et al.
Drug-Eluting and Biodegradable Stents	Metal alloys, biodegradable polymers, et al.	Combination of mechanical support and localized therapy, biodegradability eliminates need for extraction	Risk of restenosis, long-term stability of drug coating, safety of degradation products	Palliation of malignant obstructions, temporary scaffolding for benign strictures, dissolution of pancreatic duct stones, et al.

**Table 2:** Innovative application cases of three platforms.

Platform	Core Mechanism	Therapeutic Target	Key Findings	Major Challenges
**Part A: Nanoparticle (NP) Platforms**				
**A1: Lipid Nanoparticles (LNPs)**				
YTHDF1-siRNA LNP [20]	Delivers siRNA against YTHDF1 to inhibit MDSCs via the EZH2-IL-6 axis	Non-alcoholic steatohepatitis-hepatocellular carcinoma (NASH-HCC)	Enhances efficacy of anti-PD-1 therapy.	Targeting efficiency to specific cell types; complexity of modulating the immune microenvironment; long-term safety profile.
Rubicon-targeting CRISPR-Cas9 LNP [21]	Delivers CRISPR-Cas9 system to knock down Rubicon gene	Non-alcoholic fatty liver disease (NAFLD)	Improves hepatic lipid metabolism and reduces steatosis.	Potential off-target effects of gene editing; in vivo delivery efficiency; immunogenicity.
Ginger-derived biomimetic LNP for IL-22 mRNA [22]	Oral delivery platform mimicking ginger-derived nanoparticles	Ulcerative colitis	Increases IL-22 protein expression, accelerating epithelial repair.	Overcoming gastrointestinal barriers for oral delivery; stability and translational efficiency of mRNA.
**A2: Polymeric Nanoparticles (PNPs**)				
Sialyl-Tn-targeted Foretinib PNP [23]	Active targeting via anti-Sialyl-Tn antibody for tyrosine kinase inhibitor delivery	Gastric cancer	Overcomes tumor resistance and inhibits metastasis.	Heterogeneity of tumor antigen expression; batch-to-batch consistency in scalable manufacturing.
Icariin-loaded PNP [24]	Induces immunogenic cell death (ICD) to activate anti-tumor immunity	Gastric cancer	Inhibits tumor growth by up to 80% and modulates the TME.	Precise control over drug loading and release kinetics; complexity of the tumor microenvironment.
Cell-membrane-coated co-delivery PNP [25]	Cancer cell membrane coating for homologous targeting; co-delivery of Doxorubicin and Curcumin	Multidrug-resistant esophageal cancer	Enhances targeting and penetration, reverses MDR, reduces cardiotoxicity.	Complexity and reproducibility of membrane extraction and coating processes.
**A3: Inorganic Nanoparticles (INPs)**				
Multifunctional gold nanocage [26]	Photothermal therapy (PTT) synergized with anti-PD-L1 and Galunisertib	Colorectal cancer	Suppresses both primary and metastatic tumors via enhanced immunotherapy.	Long-term biosafety and clearance pathways; limited tissue penetration depth of light.
Fe_3_O_4_-based photothermal immunogenic ferroptosis agent [27]	Synergistic PTT and enzyme-catalyzed enhanced ferroptosis	Colorectal cancer	Potent tumor cell killing with integrated MRI capability.	Potential nanoparticle accumulation toxicity; quality control of complex fabrication.
Cetuximab-targeted gold nanoparticle [28]	Active EGFR targeting for co-delivery of Gemcitabine	Pancreatic ductal adenocarcinoma (PDAC)	Precise targeting of cancer and stellate cells, improving efficacy.	Poor penetration due to dense tumor stroma; cost and control of scalable production.
**Part B: In Situ-Forming Hydrogel Platforms**				
PCAC_16_ Smart Hydrogel [29]	pH-triggered charge reversal for precise antibacterial adhesion	Post-ESD wound protection/Gastric ulcer	Targets *H. pylori* and significantly promotes mucosal healing.	Retention stability and controlled degradation rate in the dynamic GI tract environment.
OXL/IL-15 Thermogel [30]	Body temperature-triggered gelation forming a local chemo-immunotherapy depot	Localized treatment of colorectal cancer	Synergistic chemo-immunotherapy with reduced systemic toxicity.	Coordinated control of release kinetics for different drugs; applicability limited to locally accessible tumors.
PCL@Mg Hydrogel Powder [31]	Instant hydration and gelation upon contact with tissue moisture via endoscopic spraying	Sutureless closure of gastric perforation	Achieves suture-free closure with anti-inflammatory and antioxidant effects.	Powder flowability and reliability of endoscopic delivery; sealing capacity for large perforations.
KGM/SA/ε-PLL Two-Component Hydrogel [32]	Rapid gelation via hydrogen bonding and electrostatic interactions after endoscopic spraying	Post-ESD wounds in esophagus and colon	Promotes epithelial proliferation, reduces inflammation, inhibits fibrosis.	Ensuring complete and uniform coverage of wounds in the peristaltic GI environment.
**Part C: Smart Stent Platforms**				
Citric acid-eluting hydrogel stent [33]	Local sustained release of citric acid to chelate calcium in stones	Minimally invasive treatment of pancreatic duct stones	Offers a novel approach for endoscopic stone dissolution.	Lack of validation in large animal models; long-term stability and biocompatibility of the coating.
Dexamethasone-eluting biodegradable biliary stent [34]	Sustained anti-inflammatory release; complete stent degradation in vivo	Benign biliary strictures (BBS)	Eliminates the need for secondary removal surgery, effective anti-fibrosis.	Matching degradation rate with tissue healing; risk of early migration.
Paclitaxel-eluting biliary metal stent [35]	Localized continuous release of paclitaxel to inhibit tumor ingrowth	Malignant biliary obstruction (MBO)	Prolongs stent patency and demonstrates local anti-tumor effects.	Balancing tumor suppression with restenosis risk; long-term stability of the drug coating.
Unidirectional biodegradable stent system [36]	Unidirectional degradation design based on single poly-L-lactic acid (PLLA) material	Biliary tract diseases	Provides flexibility in stent selection for personalized endoscopic treatment.	In vivo metabolic fate of degradation products; maintenance of mechanical properties during degradation.
Acetazolamide-eluting biodegradable anastomotic stent [37]	Local release of acetazolamide from a biodegradable stent to prevent pancreatic leakage	Pancreaticojejunal anastomotic leakage	Significantly reduces anastomotic leakage rate and promotes tissue healing.	Stent design adaptation to complex surgical anatomy; surgeon acceptance in clinical translation.

**Table 3:** Representative clinical translation cases of nano platforms.

Technology/Regimen	Payload/Drug	Clinical Trial Phase	Key Efficacy Findings	Safety Profile
NALIRIFOX Regimen [52]	Irinotecan, Oxaliplatin, Fluorouracil	Phase 3	Significant improvement in overall survival (OS) in metastatic pancreatic ductal adenocarcinoma (mPDAC)	Manageable hematological toxicity
Nanoliposomal Irinotecan (Onivyde^®^) [51]	Irinotecan	Phase 3 (Approved)	Superior OS vs. 5-FU/LV in gemcitabine-refractory mPDAC	Diarrhea, nausea, hematological toxicity
PEGylated Liposomal Irinotecan (HR070803) [54]	Irinotecan Hydrochloride	Phase 3	Significant OS benefit as first-line therapy in mPDAC	Favorable tolerability and safety
NIFTY Trial [53]	Nanoliposomal Irinotecan (nal-IRI)	Phase 2b	Improved progression-free survival (PFS) and OS in advanced biliary tract cancer (BTC)	Acceptable safety profile
COMBAT/KEYNOTE-202 [55]	Irinotecan, Motixafortide (CXCR4i), Pembrolizumab (anti-PD-1)	Phase 2	Demonstrates potential of immunomodulatory combination in gemcitabine-refractory mPC	Well-tolerated, severe neutropenia rate 7%
MCC-465 (Immunoliposome) [56]	Doxorubicin	Phase 1	PEGylated immunoliposome targeting gastric cancer cells, shows targeted delivery potential	Well-tolerated, no palmar-plantar erythrodysesthesia or cardiotoxicity
PL-MLP (Promitil^®^) [57]	Mitomycin C Lipid-based Prodrug (MLP)	Phase I/II	Long-circulating half-life, high disease stabilization rate in metastatic colorectal cancer	Well-tolerated, dose-limiting toxicity: thrombocytopenia

## Data Availability

The original contributions presented in this study are included in the article. Further inquiries can be directed to the corresponding author.

## Abbreviations

The following abbreviations are used in this manuscript:

EUS	Endoscopic Ultrasound
ESD	Endoscopic Submucosal Dissection
LNPs	Lipid Nanoparticles
PLGA	Poly(lactic-co-glycolic acid)
MSNs	Mesoporous Silica Nanoparticles
AuNPs	Gold Nanoparticles
IONPs	Iron Oxide Nanoparticles
siRNA	Small interfering RNA
mRNA	Messenger RNA
PTT	Photothermal Therapy
PDT	Photodynamic Therapy
IBD	Inflammatory Bowel Disease
NASH	Non-Alcoholic Steatohepatitis
HCC	Hepatocellular Carcinoma
NAFLD	Non-Alcoholic Fatty Liver Disease
PDAC	Pancreatic Ductal Adenocarcinoma
MDSCs	Myeloid-Derived Suppressor Cells
MUC1	Mucin 1
DES	Drug-Eluting Stents
5-FU	5-Fluorouracil
PTX	Paclitaxel
MMP	Matrix Metalloproteinase
POEM	Peroral Endoscopic Myotomy
ERCP	Endoscopic Retrograde Cholangiopancreatography
DPOC	Direct Peroral Cholangioscopy
